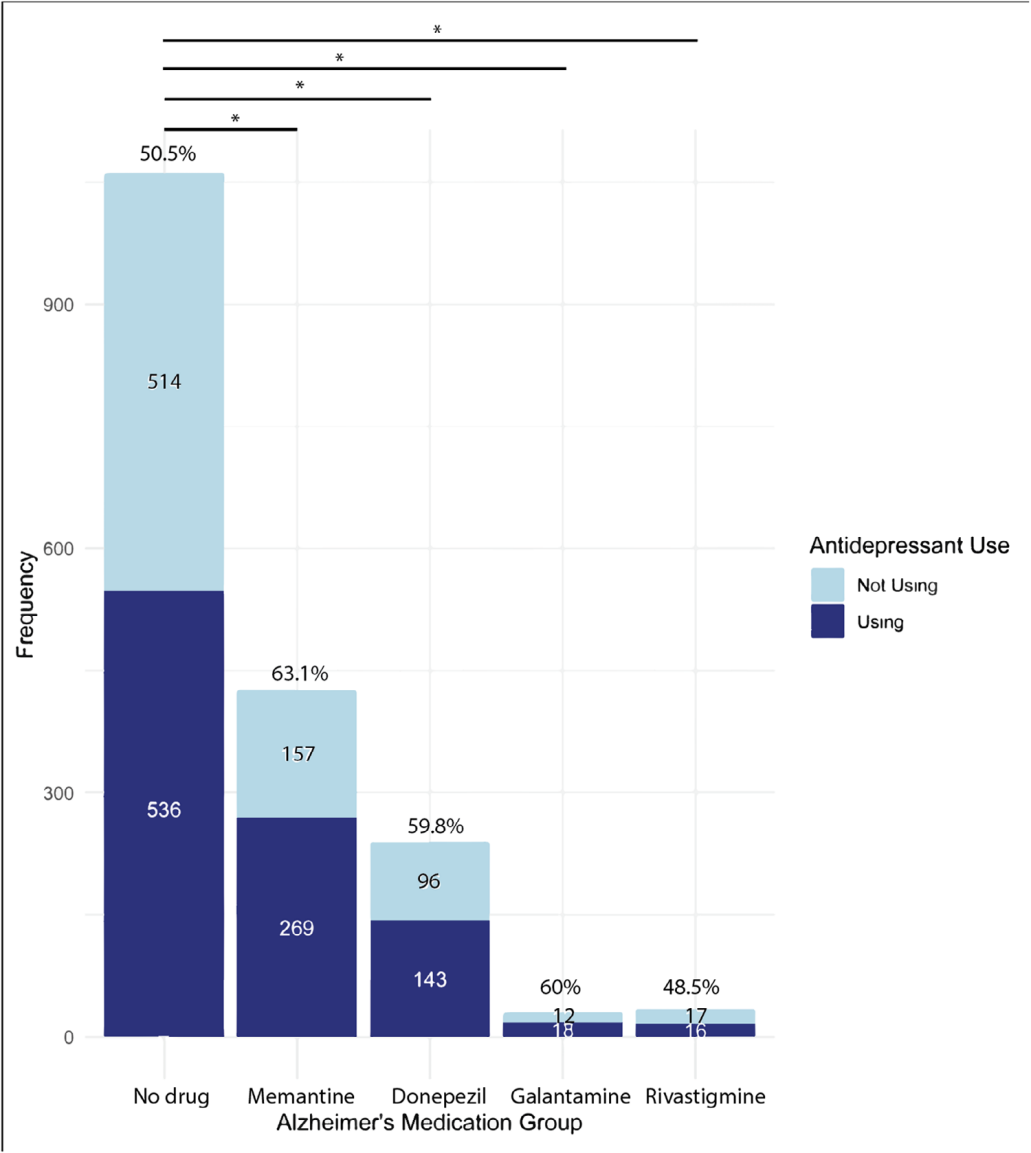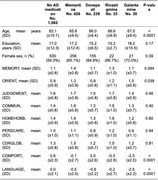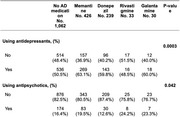# Alzheimer Disease Drugs in bvFTD: Real‐World Clinical Utilization and Behavioral Impact

**DOI:** 10.1002/alz70857_107506

**Published:** 2025-12-26

**Authors:** Tatiane Morgana da Silva, Daniel Arnold, Wyllians Vendramini Borelli, Eduardo R. Zimmer

**Affiliations:** ^1^ Neurology Department, São Lucas Hospital of PUCRS, Porto Alegre, Rio Grande do Sul, Brazil; ^2^ Universidade Federal do Rio Grande do Sul, Porto Alegre, Rio Grande do Sul, Brazil; ^3^ UFRGS, Porto Alegre, Brazil

## Abstract

**Background:**

Numerous studies have evaluated the therapeutic efficacy of acetylcholinesterase (AchE) inhibitors or memantine in frontotemporal dementia (FTD). However, some studies failed to demonstrate benefits, while others suggested potential worsening of cognitive and behavioral performance.

**Method:**

We analyzed data from the National Alzheimer's Coordinating Center (NAAC) from 2005 to 2023, focusing on participants diagnosed with behavioral variant FTD (bvFTD). We compared bvFTD patients with and without the use of AchE inhibitors (donepezil, galantamine, rivastigmine) and/or memantine, regarding the use of symptomatic AD medication. Demographic data included sex, age, and years of education. Cognitive impairment was assessed using the CDR plus NAAC FTD. Additionally, we investigated the use of behavioral medications, such as antipsychotics and antidepressants.

**Results:**

We identified approximately 1,700 participants with clinical diagnoses of bvFTD, of whom 816 (48%) reported current use of AchE inhibitors and/or memantine. A total of 613 participants were using AchE inhibitors (449 with donepezil, 73 with galantamine, and 91 with rivastigmine), and 517 were treated with memantine. Furthermore, 281 participants were using memantine in combination with at least one AchE inhibitor. The group receiving AD drugs was older, with no significant differences in education or sex, and had better scores in behavior, comportment and personality on the CDR plus NAAC FTD, although no statistical significance was observed in other domains (Table 1). Additionally, participants in this group used more antidepressants (Table 2, Figure 1).

**Conclusion:**

Our analysis showed that nearly 50% of bvFTD patients were treated with AchE inhibitors and/or memantine. Despite the lack of robust evidence supporting their efficacy in FTD, our results suggest that these medications may have a positive impact on behavior, comportment, and personality. However, participants using these drugs also required more antidepressants. These findings indicate that well‐designed studies are essential to determine effectiveness and safety of AchE inhibitors and memantine in this population.